# Prevalence of poor mental health among medical students in Nepal: a cross-sectional study

**DOI:** 10.1186/s12909-017-1083-0

**Published:** 2017-11-28

**Authors:** Arjab Adhikari, Aman Dutta, Supriya Sapkota, Abina Chapagain, Anurag Aryal, Amita Pradhan

**Affiliations:** 1 KIST Medical College and Teaching Hospital, Lalitpur, Nepal; 2 Kathmandu Medical College and Teaching Hospital, Kathmandu, Nepal

**Keywords:** Mental health, Medical students, Nepal, Depression, Anxiety, Eating disorder, Somatic symptoms, Suicidal ideation, Dropping out, Marijuana

## Abstract

**Background:**

Poor mental health among medical students is widely acknowledged. Studies on mental health among medical students of Nepal are lacking. Therefore, we conducted a study to determine the prevalence of mental disorders.

**Methods:**

A cross-sectional study was conducted among medical students at KIST Medical College and Teaching Hospital, Nepal from December 2016 to February 2017. Our survey instrument consisted of the Patient Health Questionnaire (PHQ) and questions about socio-demographic factors, smoking, marijuana use, suicidal ideation and thoughts of dropping out of medical school.

**Results:**

The prevalence rates were 29.2% (95% CI, 24.4% – 34.3%) depression, 22.4% (95% CI, 18.0% – 26.9%) medium to highly severe somatic symptoms, 4.1% (95% CI, 2.0% – 6.2%) panic syndrome, 5.8% (95% CI, 3.4% – 8.3%) other anxiety syndrome, 5% (95% CI, 2.7% – 7.3%) binge eating disorder and 1.2% (95% CI, 0.0% - 2.3%) bulimia nervosa. Sixteen students [4.7% (95% CI, 2.4% – 6.9%)] seriously considered committing suicide while in medical school. Thirty-four students [9.9% (95% CI, 6.8% – 13.1%)] considered dropping out of medical school within the past month. About 15% (95% CI, 11.1% – 18.6%) of the students reported use of marijuana during medical school.

**Conclusions:**

We found high prevalence of poor mental health among medical students of Nepal. Future studies are required to identify the factors associated with poor mental health.

**Electronic supplementary material:**

The online version of this article (10.1186/s12909-017-1083-0) contains supplementary material, which is available to authorized users.

## Background

The journey through medical school is a stressful and daunting task. High level of stress is seen in medical students [1]. Medical students at the first year of medical school have similar psychological morbidity compared to non-medical peers [2] and general population [3]. However, their mental health worsens as they course through medical school [2–4]. Despite having easy access to healthcare facilities, medical students are often reluctant to seek help for mental health concerns [5].

About one third of the medical students worldwide suffer from depression or depressive symptoms [6, 7]. Beside depression, anxiety and psychosomatic disorder [8] constitute an emerging mental health problem. Additionally, medical students are at high risk of developing eating disorders as well [9].

Poor mental health among medical students has been reported from various parts of Asia including India [10–12], Pakistan [13], Iran [14], Malaysia [15], China [16] and Saudi Arabia [4]. Poor mental health is found to be associated with serious thoughts of dropping out of medical school [17], substance abuse [18], burnout and suicidal ideation [19].

Studies on mental health of medical students in Nepal are lacking. Only few studies assessing depression [20, 21], anxiety [22] and suicidal ideation [23] were available on Pubmed, Google Scholar and Psycinfo database. Studies on other aspects of mental health including somatic symptoms and eating disorders were not available. Furthermore, the negative consequences of poor mental health including suicidal ideation, thoughts of dropping out of medical school and marijuana use are yet to be explored among Nepalese medical students. Thus, we conducted a survey to evaluate the following:

1. Prevalence of depression, somatic symptoms, anxiety syndromes and eating disorders
2. Prevalence of suicidal ideation, thoughts of dropping out of medical school and marijuana use

There are currently no programs in medical school curriculum of Nepal to screen for poor mental health or to cope with stress of being a medical student. Understanding of the mental health among medical students will encourage the development and integration of student wellness programs to prevent negative outcomes of poor mental health.

## Methods

### Study design, setting and participants

We conducted a cross-sectional survey at KIST Medical College and Teaching Hospital from December 2016 to February 2017. KIST Medical College and Teaching Hospital is affiliated to Tribhuvan University, Nepal. The medical school curriculum consists of basic science teaching in the first 2 years and clinical teaching in third, fourth and final year. The students in first 2 years have small element of clinical exposure. Final-year students were excluded from this study because of their unavailability as they were preparing for their board exams.

Our target was to include most of the students of first, second, third and fourth year. So, the questionnaire was distributed on a day with high number of attendees. Out of total 378 students, 370 were in attendance and received the questionnaire. Non-attendees and incomplete responses were excluded from the study. The study was conducted in the leisure time, in between the lectures. In a quiet classroom, after a short verbal presentation, a one-time, self-reported questionnaire was distributed. The cover letter consisted of an informed consent, which included a description of study and participants’ rights to decline altogether or leave the questions unanswered. To preserve participants’ anonymity, the questionnaire did not incorporate name, address or signature. Participants did not receive any incentives or financial compensation to participate in the study.

The total number of students in first-year and second-year was higher than third- and fourth-year students (110 and 128 vs 69 and 71 respectively) because the number of students enrolled varies annually. Nepal Medical Council determines the number of students that can be enrolled annually by a medical school. At the time of data collection, first- and second-year students had completed their first month of the academic year. Third- and fourth-year students were in their final quarter of the academic year. The study was approved by Institutional Review Committee of KIST Medical College and Teaching Hospital.

### Survey tool

Our survey instrument consisted of the Patient Health Questionnaire (PHQ) and questions about socio-demographic factors, smoking, marijuana use, suicidal ideation and thoughts of dropping out of medical school. Our survey instrument has been provided as Additional file 1 [see Questionnaire].

### PHQ

PHQ is a validated, self-administered, three page questionnaire, which assesses depression, somatic symptoms, anxiety syndromes, alcohol abuse and eating disorders [24]. In our study, depression was assessed using PHQ – 9 modules. Somatic symptom severity was assessed using PHQ – 15 modules. PHQ algorithm was used to assess panic syndrome, other anxiety syndrome, binge eating disorder and bulimia nervosa. PHQ, PHQ – 9 modules, PHQ −15 modules and PHQ algorithm (instructions) are available at http://www.phqscreeners.com/.

### Depression

PHQ requires students to rate each of the 9 depressive symptoms into ‘not at all’ , ‘several days’ , ‘more than half the days’ and ‘nearly every day’. Using PHQ – 9 modules, scores of 0, 1, 2 and 3 were assigned respectively. A cut – off score of 5, 10, 15 and 20 signifies mild, moderate, moderately severe and severe depression respectively. A score of ≥ 10 has a sensitivity of 88% and specificity of 88% for major depression [25]. In this study, students scoring ≥ 10 were considered having depression.

### Somatic symptoms

PHQ requires students to rate each of the 13 somatic symptoms into ‘not bothered at all’ , ‘bothered a little’ or ‘bothered a lot’. Using PHQ −15 modules, scores of 0, 1 and 2 were assigned respectively. A cut-off score of 5, 10, and 15 signifies low, medium and high somatic symptoms severity respectively [26]. Students scoring ≥ 10 were considered having medium to highly severe somatic symptoms.

### Panic syndrome and other anxiety syndrome

PHQ can be used to diagnose both ‘syndromes’ and ‘disorders’. However, diagnosis of ‘disorders’ from ‘syndromes’ requires further clinical interview. As our study was limited to self-administered questionnaire, our diagnosis was limited to ‘syndromes’ only.

### Smoking

Students were asked whether they had started smoking before or after joining medical school. Increment or decline in frequency of smoking was queried among students who started smoking before joining medical school.

### Marijuana use

Students were asked to quantify the frequency of marijuana use before and during medical school into no use, 1–10 times, more than 10 times but less than each month, each month but less than each week, each week but not daily and daily use [27].

### Suicidal ideation and thoughts of dropping out

To assess suicidal ideation, students were asked whether they had seriously considered committing suicide while in medical school. To assess thoughts of dropping out, students were asked whether they considered dropping out of medical school within the past month.

Positive screening for at least one syndrome identified by PHQ or having suicidal ideation was considered poor mental health in our study.

### Statistical analysis

Chi-square analysis was used to test for association between study characteristics (gender, year in medical school) and mental health problems. Statistical analysis was done using IBM SPSS Statistics 23. *P* value of < 0.05 was considered significant for statistical test.

## Results

Out of total 378 students, 370 were in attendance and received the questionnaire. Among 370 attendees, 343 (92.7%) responses were complete. Incomplete responses were excluded from the study. The response rate was 92.59%, 94.4%, 94.11% and 88.40% in first-, second-, third- and fourth-year students respectively. The following results are based on 343 complete responses.

The prevalence rates were 29.2% (95% CI, 24.4% – 34.3%) depression, 22.4% (95% CI, 18.0% – 26.9%) medium to highly severe somatic symptoms, 4.1% (95% CI, 2.0% – 6.2%) panic syndrome, 5.8% (95% CI, 3.4% – 8.3%) other anxiety syndrome, 5% (95% CI, 2.7% – 7.3%) binge eating disorder and 1.2% (95% CI, 0.0% – 2.3%) bulimia nervosa. Table 1 shows the characteristics of study population.

**Table 1:** Characteristics of the study population

Study characteristics (*n* = 343)	
Gender	
Male	175 (51%)
Female	168 (49%)
Year in medical school	
First	100 (29.2%)
Second	118 (34.4%)
Third	64 (18.7%)
Fourth	61 (17.8%)
Preclinical/Clinical	
Preclinical	218 (63.6%)
Clinical	125(36.4%)

### Depression

**Fig. 1:**
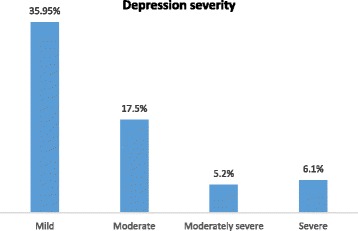
Severity of depression

The prevalence of depression was significantly higher in females compared to males (35.1% vs 23.4%; X^2^ = 5.672, *P* = 0.017). A significantly higher percentage of preclinical students reported depression compared to clinical students (33% vs 22.4%; X^2^ = 4.344, *P* = 0.037). Figure 1 shows the severity of depression.

### Somatic symptoms and severity

**Fig. 2:**
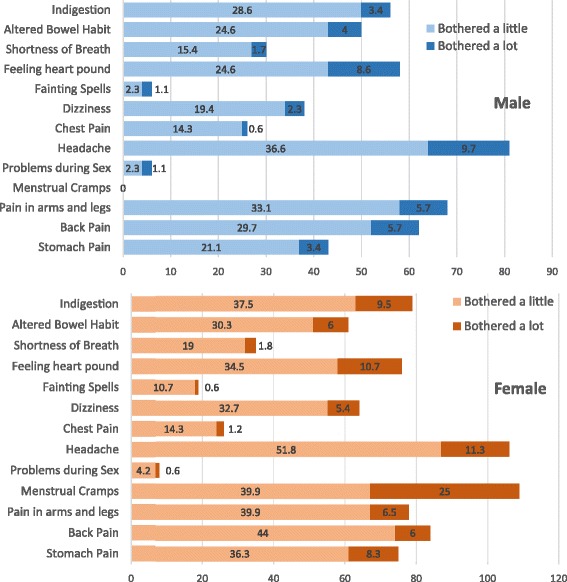
Prevalence (percentage) of somatic symptoms among medical students

A significantly higher percentage of female students reported medium to highly severe somatic symptoms (30.4% vs 14.9%; X^2^ = 11.829, *P* = 0.001). No significant difference was found between preclinical and clinical students. Figure 2 compares the frequency of somatic symptoms between male and female students. Among male students, headache was the most frequent complaint followed by pain in arms and legs. Menstrual cramps followed by headache were the most commonly reported complaints among female students.

### Anxiety syndromes

The prevalence rates were panic syndrome, 5.8% (95% CI, 3.4% – 8.3%) and other anxiety syndrome, 5% (95% CI, 2.7% – 7.3%). No significant difference was found between male and female students as well as between preclinical and clinical students.

### Suicidal ideation

Sixteen students (4.7% [95% CI, 2.4% – 6.9%]) seriously considered committing suicide while in medical school. No significant difference was found between male and female students. Students in clinical years were more likely to report suicidal ideation compared to preclinical students (8% vs 2.8%; X^2^ = 4.920, *P* = 0.027). A significantly higher percentage of depressed students reported suicidal ideation compared to students without depression (12% vs 1.6%; X^2^ = 17.078, *P* = 0.000).

### Thoughts of dropping out

Thirty-four students (9.9% [95% CI, 6.8% – 13.1%]) considered dropping out of medical school within the past month. No significant difference was found between male and female students as well as between preclinical and clinical students. Students with depression were more likely to have thoughts of dropping out compared to students without depression (19% vs 6.2%; X^2^ = 13.053, *P* = 0.000).

### Eating disorder

Binge eating disorder was present in 5% (95% CI, 2.7% – 7.3%) of the students. About 10% of the students with depression were also positive for binge eating disorder. This relation was statistically significant (X^2^ = 7.997, *P* = 0.005). No significant difference was found between male and female students.

### Smoking

The prevalence of smoking among medical students was 14.3% (95% CI, 10.6% – 18.0%). The prevalence of smoking was significantly higher in males compared to females (26.3% vs 1.8%; X^2^ = 42.018, *P* = 0.000). Majority of students (69%) started smoking before joining medical school. Among them, 47% reported an increase in frequency of smoking after joining medical school whereas 53% of them reported decrease in smoking frequency.

### Marijuana use

About 15% [95% CI, 2.7% – 7.3%] of the students reported use of marijuana during medical school. Table 2 shows the frequency of marijuana use before and during medical school. Before joining medical school 3.49% of students had used marijuana 10 times or more whereas 5.83% of the students used marijuana 10 times or more during medical school. Marijuana use during medical school was significantly higher in male students than in female students (24.6% vs 4.8%; X^2^ = 26.573, *P* = 0.000). Students with depression were no more likely than students without depression to report marijuana use (15% vs 14.8%; X^2^ = 0.002, *P* = 0.965). Similarly, we found no significant association between other mental disorders (medium to highly severe somatic symptoms, panic syndrome and other anxiety syndrome) and marijuana use.

**Table 2:** Frequency of marijuana use before and during medical school

Frequency	Before medical school	During medical school
No use	314	292
1–10 times	17	31
More than 10 times but less than each month	5	2
Each month but less than each week	1	5
Each week but not daily	3	8
Daily use	3	5

## Discussion

The aim of our study was to determine the prevalence of various mental disorders among medical students of Nepal. In Nepal, the prevalence of depression among general population of age group 18–25 years is 2.5% [28]. In our study, we found the prevalence to be 29.2% which is higher than the general population. However, the prevalence in general population was determined using hospital anxiety and depression scale compared to PHQ used in our study. Our finding is comparable with recent meta-analysis studies 27.2% [7], 28% [6] as well as previous studies from Nepal 29.8% [20], 29.9% [22].

The current study found that 22.4% of students had medium to highly severe somatic symptoms. This percentage is high compared to medical students in Germany, where the prevalence is about 15% [29]. Our finding supports the idea that psychosomatic disorder is an emerging mental health problem among medical students [8].

We found the prevalence of panic syndrome and other anxiety syndrome to be 4.1% and 5.8% respectively. Using same questionnaire, the prevalence of panic syndrome and other anxiety syndrome in German medical students was 4.4% and 1.9% respectively [29]. Using Depression Anxiety and Stress Scale, Kunwar et al. [22] found 41.1% of the students in two medical colleges of Nepal had anxiety disorder. This difference may be due to different study instrument used.

In this study, the prevalence of eating disorders was 5% binge eating and 1.2% bulimia nervosa. There was a significant positive correlation between depression and binge eating disorder. These results match those observed in earlier studies [9, 30].

According to a recent meta-analysis by Rotenstein et al., the pooled prevalence of suicidal ideation among medical students is 11.1% [7]. The results of this study show a prevalence of 4.7%. When compared to the same meta-analysis, an interesting finding was that the prevalence of depression was higher in our study but the prevalence of suicidal ideation was lower. This finding is difficult to explain. Differences in socio-cultural aspects may be responsible. A prospective study showed that suicidal ideation during medical school predicted postgraduate suicidal ideation [19]. Such thoughts persists into practice as well [31]. So, it is necessary to effectively deal with such thoughts in time.

To our knowledge, this is the first study to report thoughts about dropping out and marijuana use among Nepalese medical students. Our result was similar to a multi-institutional study in US [17], which reported 11% of the students had thoughts of dropping out. Marijuana is second to alcohol as the most common substance to be abused by medical students [32]. It is more commonly used to ‘feel good’ and ‘have a good time’ [32] than as a coping strategy to stress [33].The reason for marijuana use among Nepalese medical students should be addressed in future studies. We hope that our findings will provide future researchers with comparative data.

### Gender

Females were more likely to have medium to highly severe somatic symptoms and depression. Previous studies report varying relationship between female gender and depression. Some studies [10, 13] show higher prevalence in female students while other studies [11, 12, 14] show no difference. High prevalence of depression seen in female medical students has been linked to personality traits [34], gender inequity and associated stigma [35].

### Year in medical school

Prevalence of depression was significantly higher in preclinical students. Similarly, studies from Pakistan [13] and India [11] report higher prevalence of depression in preclinical students. Longitudinal studies [3, 36] done in American medical students have shown high prevalence of depression in second year of medical school. The observed high rate of depression in second-year students in our study may be due to academic burnout. Students must pass the second year-university exams in first two attempts. Otherwise, they will have to face a delay of 1 year before attempting again. Similarly, human cadaver dissection which is done in preclinical year is stressful for many students [37]. Depression in first-year students may be due to stress of having to face a new challenging environment [38]. In Nepal, most students enroll into medical college directly from high school, compared to US students who enter medical school with a bachelor degree [39]. This may cause an abrupt increase in academic burden, leading to stress among first-year students.

### Recommendations

Future studies are required to identify and explore the factors associated with poor mental health among Nepalese medical students. There are currently no programs to identify and help students with their mental health and substance abuse problems. Various solutions to reduce stress like creating a nurturing learning environment, identifying and assisting students, teaching skills for stress management and promoting self-awareness have been proposed [38]. Integration of such programs as a part of medical education with special focus on preclinical students and female students may improve mental health among students.

### Limitations

Samples in this study were from a single medical school. As the study was conducted in the classroom, students were in close seating arrangement which may have affected students’ privacy leading to under representation of results of smoking and marijuana use.

## Conclusions

We found high prevalence of poor mental health among medical students of Nepal. Future studies are required to identify the factors associated with poor mental health.

## Additional file

Additional file 1: Survey instrument. Contains questionnaire of the study. (PDF 831 kb)

## Abbreviations

CI: Confidence interval
DSM – IV: Diagnostic and Statistical Manual of mental disorders – IV
PHQ: Patient Health Questionnaire
