# Supplementary material for: Prevalence of poor mental health among medical students in Nepal: a cross-sectional study
Source: BMC Med Educ. 2017 Nov 28;17:232. doi: 10.1186/s12909-017-1083-0 (PMC5704530; doi:10.1186/s12909-017-1083-0)
Supplement: Additional file 1: — Survey instrument. Contains questionnaire of the study. (PDF 831 kb) [file 12909_2017_1083_MOESM1_ESM.pdf]

MENTAL HEALTH AMONG MEDICAL STUDENTS OF NEPAL

CONSENT FORM

Dear Participant,

We invite you to participate in a research study entitled 'Mental Health among Medical Students of Nepal.' The purpose of the research is to determine the prevalence of mental disorders among medical students. The enclosed questionnaire has been designed to collect information on symptoms of somatoform disorder, depression, anxiety, eating disorder, smoking and Marijuana use.

Your participation in this research project is completely voluntary. You may decline altogether, or leave blank any questions you don't wish to answer. There are no known risks or discomforts associated with this survey. Your responses will be kept strictly confidential, and digital data will be stored in secure computer files after it is entered. Data from this research will be reported only as a collective combined total. Any report of this research that is made available to the public will not include your name or any other individual information by which you could be identified.

If you agree to participate in this project, please answer the questions on the questionnaire as best you can. It should take approximately 15 minutes to complete. Please return the questionnaire as soon as possible. If you have any questions about this project, feel free to contact Arjab Adhikari at docarjab@gmail.com. Information on the rights of human subjects in research is available through the Institutional Review Board of KIST Medical College Teaching Hospital. Email: irbkistmc@gmail.com
Completing this survey indicates that you are 18 years of age or older and indicates your consent to participate in the research.

Thank you for your assistance in this important endeavor.

Sincerely yours,

Arjab Adhikari, MBBS (Principal investigator)

How Are We Doing?

Please take a few minutes to fill out this survey. Your answers will be kept confidential. Thank you for your participation.

Socio-Demographic Information

Age :

Gender: ☐ Male ☐ Female

Year of Medical School: ☐ First ☐ Second ☐ Third ☐ Fourth

Where do you stay?: ☐ At Home ☐ Rented Room ☐ Hostel ☐ Others (Specify)_______________

Personal Information

Relationship Problem in family/friends: ☐ Yes ☐ No

Mental Disorders in family: ☐ Yes ☐ No

Dissatisfaction with your Academic Performance: ☐ Yes ☐ No

High parental expectations: ☐ Yes ☐ No

Dissatisfaction with career choice: ☐ Yes ☐ No

Lack of opportunities for leisure activities: ☐ Yes ☐ No

PATIENT HEALTH QUESTIONNAIRE (PHQ)

1. During the <u>last 4 weeks</u>, how much have you been bothered by any of the following problems?	Not bothered	Bothered a little	Bothered a lot
**a.** Stomach pain	☐	☐	☐
**b.** Back pain	☐	☐	☐
**c.** Pain in your arms, legs, or joints (knees, hips, etc.)	☐	☐	☐
**d.** Menstrual cramps or other problems with your periods	☐	☐	☐
**e.** Pain or problems during sexual intercourse	☐	☐	☐
**f.** Headaches	☐	☐	☐
**g.** Chest pain	☐	☐	☐
**h.** Dizziness	☐	☐	☐
**i.** Fainting spells	☐	☐	☐
**j.** Feeling your heart pound or race	☐	☐	☐
**k.** Shortness of breath	☐	☐	☐
**l.** Constipation, loose bowels, or diarrhea	☐	☐	☐
**m.** Nausea, gas, or indigestion	☐	☐	☐

2. Over the <u>last 2 weeks</u>, how often have you been bothered by any of the following problems?	Not at all	Several days	More than half the days	Nearly every day
**a.** Little interest or pleasure in doing things	☐	☐	☐	☐
**b.** Feeling down, depressed, or hopeless	☐	☐	☐	☐
**c.** Trouble falling or staying asleep, or sleeping too much	☐	☐	☐	☐
**d.** Feeling tired or having little energy	☐	☐	☐	☐
**e.** Poor appetite or overeating	☐	☐	☐	☐
**f.** Feeling bad about yourself — or that you are a failure or have let yourself or your family down	☐	☐	☐	☐
**g.** Trouble concentrating on things, such as reading the newspaper or watching television	☐	☐	☐	☐
**h.** Moving or speaking so slowly that other people could have noticed? Or the opposite — being so fidgety or restless that you have been moving around a lot more than usual	☐	☐	☐	☐
**i.** Thoughts that you would be better off dead or of hurting yourself in some way	☐	☐	☐	☐

3. Questions about anxiety.

		NO	YES
**a.**	In the last 4 weeks, have you had an anxiety attack — suddenly feeling fear or panic?	☐	☐

If you checked "NO", go to question #5.

		NO	YES
**b.**	Has this ever happened before?	☐	☐
**c.**	Do some of these attacks come suddenly out of the blue — that is, in situations where you don't expect to be nervous or uncomfortable?	☐	☐
**d.**	Do these attacks bother you a lot or are you worried about having another attack?	☐	☐

4. Think about your last bad anxiety attack.

		NO	YES
**a.**	Were you short of breath?	☐	☐
**b.**	Did your heart race, pound, or skip?	☐	☐
**c.**	Did you have chest pain or pressure?	☐	☐
**d.**	Did you sweat?	☐	☐
**e.**	Did you feel as if you were choking?	☐	☐
**f.**	Did you have hot flashes or chills?	☐	☐
**g.**	Did you have nausea or an upset stomach, or the feeling that you were going to have diarrhea?	☐	☐
**h.**	Did you feel dizzy, unsteady, or faint?	☐	☐
**i.**	Did you have tingling or numbness in parts of your body?...	☐	☐
**j.**	Did you tremble or shake?	☐	☐
**k.**	Were you afraid you were dying?	☐	☐

5. Over the <u>last 4 weeks</u>, how often have you been bothered by any of the following problems?

		Not at all	Several days	More than half the days
**a.**	Feeling nervous, anxious, on edge, or worrying a lot about different things.	☐	☐	☐

If you checked "Not at all", go to question #6.

		Not at all	Several days	More than half the days
**b.**	Feeling restless so that it is hard to sit still.	☐	☐	☐
**c.**	Getting tired very easily.	☐	☐	☐
**d.**	Muscle tension, aches, or soreness.	☐	☐	☐
**e.**	Trouble falling asleep or staying asleep.	☐	☐	☐
**f.**	Trouble concentrating on things, such as reading a book or watching TV.	☐	☐	☐
**g.**	Becoming easily annoyed or irritable.	☐	☐	☐

		NO	YES
**6. Questions about eating.**			
**a.**	Do you often feel that you can't control what or how much you eat?	☐	☐
**b.**	Do you often eat, within any 2-hour period, what most people would regard as an unusually large amount of food?	☐	☐
**If you checked "NO" to either #a or #b, go to question #9.**			
**c.**	Has this been as often, on average, as twice a week for the last 3 months?	☐	☐

		NO	YES
**7. In the last 3 months have you often done any of the following in order to avoid gaining weight?**			
**a.**	Made yourself vomit?	☐	☐
**b.**	Took more than twice the recommended dose of laxatives?	☐	☐
**c.**	Fasted — not eaten anything at all for at least 24 hours?	☐	☐
**d.**	Exercised for more than an hour specifically to avoid gaining weight after binge eating?	☐	☐

	NO	YES
**8. If you checked "YES" to any of these ways of avoiding gaining weight, were any as often, on average, as twice a week?**	☐	☐

	NO	YES
**9. Do you ever drink alcohol (including beer or wine)?**	☐	☐
**If you checked "NO" go to question #11.**		

		NO	YES
**10. Have any of the following happened to you more than once in the last 6 months?**			
**a.**	You drank alcohol even though a doctor suggested that you stop drinking because of a problem with your health.	☐	☐
**b.**	You drank alcohol, were high from alcohol, or hung over while you were working, going to school, or taking care of children or other responsibilities.	☐	☐
**c.**	You missed or were late for work, school, or other activities because you were drinking or hung over.	☐	☐
**d.**	You had a problem getting along with other people while you were drinking.	☐	☐
**e.**	You drove a car after having several drinks or after drinking too much.	☐	☐

11. If you checked off any problems on this questionnaire, how difficult have these problems made it for you to do your work, take care of things at home, or get along with other people?

Not difficult at all	Somewhat difficult	Very difficult	Extremely difficult
☐	☐	☐	☐

Developed by Drs. Robert L. Spitzer, Janet B.W. Williams, Kurt Kroenke and colleagues, with an educational grant from Pfizer Inc. No permission required to reproduce, translate, display or distribute.

About Smoking

Do you Smoke: ☐ Yes ☐ No If you check "**NO**" then continue to next block

When did you start smoking?

☐ Before Joining Medical School ☐ After Joining Medical School

If you started before joining medical school, did it increase or decrease after joining medical school?

☐ Increase ☐ Decrease

About Marijuana use

Before Medical School

- ☐ No use
- ☐ 1 – 10 times
- ☐ More than 10 times but less than each month
- ☐ Each month but less than each week
- ☐ Each week but not daily
- ☐ Daily

During Medical School

- ☐ No use
- ☐ 1 – 10 times
- ☐ More than 10 times but less than each month
- ☐ Each month but less than each week
- ☐ Each week but not daily
- ☐ Daily

Suicidal Ideation

Have you seriously considered committing suicide while in medical school? ☐ Yes ☐ No

Dropping Out

Have you considered dropping out of medical school during past month? ☐ Yes ☐ No

Thank you for completing our survey.
